# Cross-sectional study of the prevalence of prescription opioids misuse in French patients with chronic non-cancer pain: An update with the French version of the POMI scale

**DOI:** 10.3389/fphar.2022.947006

**Published:** 2022-09-05

**Authors:** Nicolas Kerckhove, Noémie Delage, Célian Bertin, Emmanuelle Kuhn, Nathalie Cantagrel, Caroline Vigneau, Jessica Delorme, Céline Lambert, Bruno Pereira, Chouki Chenaf, Nicolas Authier, Poma Network, Debbah Abdelouahab

**Affiliations:** ^1^ Service de Pharmacologie Médicale, Centres Addictovigilance et Pharmacovigilance, Centre d’Evaluation et de Traitement de la Douleur, Inserm, CHU Clermont-Ferrand, Neuro-Dol, Université Clermont Auvergne, Clermont-Ferrand, France; ^2^ Institut Analgesia, Université Clermont Auvergne, Clermont-Ferrand, France; ^3^ Observatoire Français des Médicaments Antalgiques (OFMA), CHU Clermont-Ferrand, Université Clermont Auvergne, Clermont-Ferrand, France; ^4^ Centre d'évaluation et d’information sur la Pharmacodépendance—Addictovigilance, CHU Nantes, Nantes, France; ^5^ Centre d’Evaluation et de Traitement de la Douleur, CHU Toulouse, Toulouse, France; ^6^ Unité de Biostatistiques, DRCI, CHU de Clermont-Ferrand, Clermont-Ferrand, France

**Keywords:** opioids, prevalence, chronic non-cancer pain, POMI scale, prescription drug misuse

## Abstract

Public health issues related to chronic pain management and the risks of opioid misuse and abuse remain a challenge for practitioners. Data on the prevalence of disorders related to the use of prescribed opioids in patients suffering from chronic pain remains rather patchy, in particular because of the absence of a gold standard for their clinical assessment. We estimated the prevalence of prescription opioid misuse (POM), using a specific and validated opioid misuse scale (POMI-5F scale), in adults with chronic non-cancer pain. Nine-hundred-fifty-one (951) patients with opioids prescription and followed-up in pain clinics and addictology centers for chronic non-cancer pain (CNCP) completed the survey interview. The results suggest that 44.4% of participants have POM, accompanied by overuse (42.5%), use of opioids for effects other than analgesia (30.9%), withdrawal syndrome (65.7%), and craving (6.9%). The motivations cited for POM, apart from pain relief, were to calm down, relax and improve mood. POM was shown to be related to male sex (OR 1.52), young age (OR 2.21) and the presence of nociplastic pain (OR 1.62) of severe intensity (OR 2.31), codeine use (OR 1.72) and co-prescription of benzodiazepines (OR 1.59). Finally, despite the presence of three subgroups of misusers, no factor was associated with the intensity of misuse, reinforcing the view that distinguishing between strong and weak opioids is not appropriate in the context of use disorder. Almost half of patients with CNCP misuse their prescribed opioid. Practitioners should be attentive of profiles of patients at risk of POM, such as young, male patients suffering from severe nociplastic pain, receiving prescription for codeine and a co-prescription for benzodiazepine. We encourage French-speaking practitioners to use the POMI-5F scale to assess the presence of POM in their patients receiving opioid-based therapy.

**Clinical Trial Registration** clinicaltrials.gov, identifier NCT03195374

## Introduction

Chronic pain is a common problem, affecting approximately 20%–31% of the general adult population, depending on the country (Steingrímsdóttir et al., 2017). The management of chronic pain frequently involves the use of analgesics, with no real pharmacological innovation in recent years. Conversely, in recent years there has been growing concern about the appearance of certain complications specifically related to opioid analgesics. These complications raise questions about their real benefit/risk balance in certain indications, due to the risk of iatrogenic physical dependence, opioid use disorder (OUD), overdose and related death that they can induce. In this regard, the opioid crisis affecting several countries, in particular North America, illustrates a very delicate situation. Proper use of opioids is therefore essential in order to avoid an imbalance in the benefit/risk balance.

The pharmacological class of opioid analgesics includes all the natural and synthetic derivatives of the poppy, of which morphine is the foremost. They have in common that they bind to opioid receptors, the source of both their undesirable and desired effects. Their usual indications are moderate-to-severe nociceptive pain (post-traumatic, per- and postoperative, cancer pain and hyperalgesic paroxysms). Due to their several adverse effects (constipation, nausea, vomiting, sedation, dyspeptic effects, respiratory depression, physical dependence, opioid use disorder and overdose) and doubts about their efficacy against chronic non-cancer pain (CNCP), their long-term administration should not be recommended (Noble et al., 2010; Els et al., 2017). Nevertheless, their use in CNCP was initially supported by Portenoy and Foley (Portenoy and Foley, 1986). Shortly thereafter, a United Kingdom study concluded that 62% of physicians prescribed opioids in CNCP, mainly because of the failure of other treatments (Coniam, 1989). Moreover, most of the physicians expressed little concern about physical dependence and OUD as barriers to opioid prescription. This incentive to prescribe opioids has continued and led in part to the so-called “opioid crisis.” Currently, the adequate prescription of opioids in patients with CNCP is currently described in the guidelines of the Centers for Disease Control and Prevention (Dowell et al., 2016) and the European Pain Federation (Häuser et al., 2021).

Based on reports of Analgesic, Anesthetic, and Addiction Clinical Trials, Translations, Innovations, Opportunities, and Networks (Smith et al., 2013) and the Initiative on Methods, Measurement, and Pain Assessment in Clinical Trials (O’Connor et al., 2013), three definitions are used to categorize problematic use as addiction, misuse, or abuse. Semantic confusion exists between addiction and misuse by physicians not specialized in addictology, which was increased with the publication of the 5th edition of the Diagnostic and Statistical Manual of Mental Disorders (DSM-5) which brings all these notions together under the term “substance use disorder,” described as a continuum between different alternative substance uses (abuse, misuse, addiction). This semantic confusion is still the subject of much debate and can lead to the stigmatization of chronic pain patients treated with opioid analgesics (Bertin et al., 2021). It is therefore important to clarify the diagnostic approach in this field. Individuals misusing opioids include those with 1) undertreated chronic pain receiving prescription opioids; 2) vulnerability to opioid misuse and who are exposed to opioids during an acute pain episode; and 3) use opioids for non-medically supervised recreational use or relief from psychological and/or emotional stress (Cicero and Ellis, 2017; Stumbo et al., 2017).

According to a review by Vowles, among the US patients suffering from chronic pain and taking opioids, about 21%–29% developed a misuse (Vowles et al., 2015). A French study highlighted a higher prevalence with 52% of opioids misuse, assessed by DSM-5 criteria (Eiden et al., 2019). Another recent French study found 43.3% of patients with CNCP had current moderate or severe DSM-5 criteria (Guillou-Landreat et al., 2021). However, these studies used the DSM-5 dimensional diagnosis of substance use disorder to assess misuse. The DSM-5 clinical criteria have not been validated in French and are not specific to misuse alone as they are also used to assess addiction. These limitations may lead to confusion and misestimating the true rate of misuse and addiction (Kaye et al., 2017). This confusion can have a clinical impact, as the specificities of managing misuse and addiction differ from each other. The Computerized Current Opioid Misuse Measure (COMM) scale exists to assess misuse, but it is used very little or not at all in the clinic because of its length and is mainly used for research (Butler et al., 2007). Therefore, the Prescription Opioid Misuse Index (POMI) scale is the only one that specifically assesses clinical misuse (Knisely et al., 2008); it has also recently been translated into French and validated by our team (Delage et al., 2022). Thus, the primary objective of the present study was to update and to assess the prevalence of opioid misuse in French patients suffering from CNCP and followed-up in pain clinics and addictology centers, using the POMI scale, and to characterize chronic pain patients with Prescription Opioid Misuse (POM). The secondary objectives were: 1) to investigate the relationship between the presence of POM and type of opioids; 2) to characterize misuser profiles and the modalities of POM; and 3) to identify factors associated with the presence of POM.

## Materials and methods

### Ethical and regulatory aspects

The study was approved by the Research Ethics Committee (*Comité de Protection des Personnes Sud-Est VI, Clermont-Ferrand, France*) on 21 July 2016 (ref: AU1263), the French personal data protection authority (*Comité Consultatif sur le Traitement de l’Information en matière de Recherche dans le domaine de la Santé, ref. 16-643*) and registered on Clinicaltrials.gov (NCT03195374). The research was conducted in accordance with the World Medical Association Declaration of Helsinki (Tokyo 2004; revised).

Study data were managed using REDCap (Harris et al., 2009) electronic data capture tools hosted by the sponsor of the study (University Hospital of Clermont-Ferrand, France). The REDCap web platform (http://project-redcap.org/) possesses the authorization of the French personal data protection authority.

### Study design and population

This cross-sectional and multicenter study was offered to all adult patients suffering from CNCP for at least 6 months, treated by opioids for at least 3 months, and followed-up in pain clinics and addictology centers. Only patients with sufficient comprehension of French to answer the questionnaires and who volunteered to participate were included in the study.

The study was systematically proposed to eligible patients during an addictology or chronic pain consultation as part of their usual care with the investigator. An information form was given to each patient, summarizing the objectives of the study and the strictly confidential aspect of the treatment of their medical data. Patients were free to accept or not to participate in this study, without impact on the quality of care and the relationship with their physician in the case of refusal. In the case of acceptance of participation, the patients had to fill in the paper form (including all the questionnaires) on site and return it to the investigator. At the end of the study, all the patient forms were sent by mail to the University Hospital of Clermont-Ferrand and the data were entered twice and compared by two clinical research assistants in the REDCap database for statistical analysis.

### Study outcomes

**FIGURE 1:**
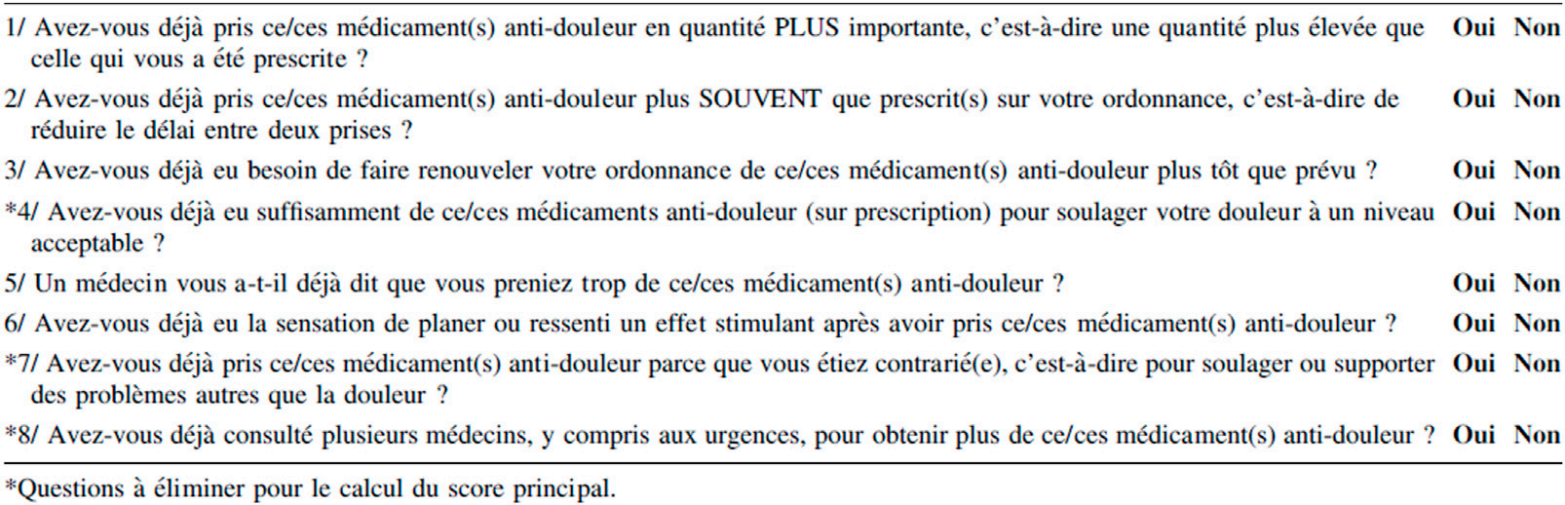
French version of POMI scale (POMI-5F) according to Delage et al., 2022.

The primary objective of the study was to estimate the prevalence of POM in a population of patients with CNCP followed-up in pain clinics and addictology centers. To achieve this objective, the POMI scale, previously translated and validated in French for 154 patients (Delage et al., 2022), was used Figure 1. The French version of the POMI scale (POMI-5F) is a self-evaluation scale with five items scored 0 (absence) or 1 (presence) that are summed to calculate a score. When this score reaches two points, the patient is considered positive and described as POM.

The secondary objectives were: 1) to investigate the relationship between the presence of POM and type of opioids; 2) to characterize misuser profiles and the modalities of POM; and 3) to identify factors associated with the presence of POM. To address our secondary objectives, a home-made questionnaire (not validated, but including several items from validated questionnaires, such as Brief Pain Inventory and DSM-5) describing all analgesic treatments (including opioids), patient sociodemographic data, clinical data [pain type, intensity (11-point numerical rating scale) and duration, and associated comorbidities], misuse and associated behavior (withdrawal syndrome, craving and opioid use for non-analgesic effects) was completed.

A withdrawal syndrome was confirmed when the patient answered “YES” to at least one of the possible answers to the following question: Have you ever experienced one or more of these symptoms when your opioid medication(s) was/were delayed or in the hours before usual intake or when the dose was decreased or when it was stopped suddenly? (multiple responses possible: Yawning; Sweating; Weeping eyes; Runny nose; Hot flashes; Joint/muscle pain; Restlessness/Anxiety; Cramps in muscles; Feeling sick/Nausea; Stomach ache; Goose bumps; Shaking chills).

Craving was confirmed when the patient answered “YES” to the following question: Have you ever felt an uncontrollable urge to use your opioid medication(s) in a non-painful setting? Finally, use for other effects (non-analgesic) was analyzed with at least one “YES” response to the following question: Have you ever taken this/these pain medication(s) for the following reasons? (multiple responses possible: To be less anxious; To calm down/soothe yourself; To improve your mood/Be less sad; Stimulate yourself/Wake up; Relax; Sleep/Fall asleep; Euphoria/Pleasure).

### Statistics

This cross-sectional study conducted on a sample should make it possible to generalize the results to the entire target population. A margin of error on the estimate is defined to estimate the number of participants required, in addition to the expected proportion of chronic pain subjects with misuse. Thus, according to the literature, for an expected proportion of misuse subjects of about 40% (Vowles et al., 2015; Guillou-Landreat et al., 2021), the inclusion of at least 577 subjects was necessary to obtain an accuracy for this proportion equals to ±4%. For 1,025 subjects this accuracy was ±3%. Thus, the inclusion of at least 800 participants was a good compromise regarding the accuracy and the feasibility of the study.

Categorical parameters were expressed as frequencies and associated percentages, and continuous data as mean ± standard deviation or as median (25th; 75th percentiles), according to statistical distribution. The assumption of normality (Gaussian) distribution was assessed using the Shapiro-Wilk test.

The prevalence of a POMI-5F score ≥2 of patients with CNCP was presented with a 95% confidence interval (CI) calculated by an exact binomial distribution. The comparisons according to the POMI-5F score (<2 versus ≥2, i.e., negative versus positive) were conducted using Chi-squared or Fisher’s exact tests for categorical data. For continuous data, the comparisons were performed with Student’s *t*-test or the Mann-Whitney test if the assumptions of the *t*-test were not met. The equality of variances was analyzed by the Fisher-Snedecor test. The relationships between POMI-5F score (<2 versus ≥2) and opioid use for non-analgesic effects and type of withdrawal symptoms were also described with the aforementioned statistical tests.

Then, in order to determine factors associated with the POMI-5F score ≥2, a multivariable logistic regression model was run using the stepwise approach (backward and forward) on covariates fixed according to univariate results and to clinical relevance. Particular attention has been paid to the study of multicollinearity and interactions between covariates: 1) studying the relationships between the covariates, and 2) assessing the impact of adding or deleting variables in the multivariable model. Furthermore, variance inflation factor, a measure of the amount of multicollinearity, was also calculated with collin command from Stata. The results were expressed as odds-ratios and 95% CI.

Finally, to determine patterns on patients at risk of POM, a multiple correspondence analysis (MCA) followed by a mixed unsupervised classification (k-means clustering applied to the partition obtained from an ascending hierarchical classification using Ward’s distance) were proposed to: 1) study the relations between the modalities of the variables, and 2) determine the profiles of the participants (groups of individuals sharing very similar characteristics). MCA can be used to perform cross tabulations and can be considered as a useful tool for revealing the relationships among categorical variables. The significance of association as tested by Chi-squared or Fisher’s exact tests provides no information regarding significant individual associations between row-column pairs. On the contrary, factorial analyses such as MCA show how the variables are related, and not just if a relationship exists. This approach enables handling a large amount of data in order to unfold hidden patterns. For these analyses, the variables were chosen according to univariate results, clinical relevance and statistical distribution (parameters always present or always absent were not considered). Only individuals without missing data were included in this analysis.

Statistical analyses were performed using Stata software (version 15, StataCorp, College Station, Texas, United States) and R 3.3.3 (http://cran.r-project.org/). All the tests were two-sided, with a Type I error set at 0.05. Particular attention was given to the magnitude of differences expressed with effect-sizes, in addition to inferential statistical tests expressed using *p*-values without Type I error correction.

## Results

### Population

The study was proposed to 1,801 patients with CNCP, and 960 were included for the study in 44 French pain clinics and addictology centers (841 refused to participate and nine were excluded because they did not have an opioid prescription). Nine hundred and fifty-one patients were analyzable for the primary objective. The first patient was enrolled on 24 August 2018, and the last patient on 09 July 2019.

Participating patients were mostly middle-aged women, in a couple and having a professional activity. Among patients in professional activities, 16.3% were in work stoppage and 42.6% were in disability due to their CNCP. Moreover, a majority of patients elicited comorbidities such as sleep and anxiety disorders for about half of them while 29.0% of them had depressive disorders.

Regarding the characterization of pain disorders and their opioid treatments, patients suffered mainly from nociplastic pain (64.0%), with a duration longer than 5 years for more than 60.0% of them, and most of them (about 90.0%) had a moderate to severe pain intensity. Finally, opioid treatments used by patients were mainly tramadol (44.2%) followed by oxycodone (20.0%). The median percentage of pain relief by opioids declared by the patient, was 50.0%. In parallel, other concomitant analgesic treatments were prescribed (see Table 1 for details), and 38.4% of patients used benzodiazepines. Finally, more than half of patients declared withdrawal symptoms related to their opioids, about 40% declared using their opioids with a higher dosage than that prescribed, about 30% reported using their opioids for non-analgesic effects, and less than 10% had a craving behavior.

**TABLE 1:** Study population characteristics. Data of all included patients (*n* = 951) are presented as percentages, mean ± standard deviation or median (25th; 75th percentiles). * Dosage or frequency higher than prescribed.

Sociodemographic characteristics	
Female sex	68.9
Age (years)	51.8 ± 13.0
Single	34.6
Professional situation	
Student	1.2
Unemployed	11.2
Retired	20.5
Employed	67.1
In activity	41.0
Work stoppage	16.3
Disability	42.6
Pain characteristics	
Type of pain	
Neuropathic	45.4
Nociplastic	64.0
Nociceptive	31.6
Pain duration	
6–12 months	2.6
1–5 years	34.5
>5 years	62.9
Pain intensity (/10)	5.9 ± 1.8
Mild (0–3)	10.2
Moderate (4–6)	51.6
Severe (7–10)	38.2
Pain relief by treatment (%)	50 [40; 70]
Treatments	
Opioids	
Morphine	14.6
Fentanyl	7.4
Oxycodone	20.0
Hydromorphone	0.6
Tramadol	44.2
Codeine	14.6
Dihydrocodeine	2.1
Opium	12.6
Number of concomitant opioids	
1	85.2
2	13.8
≥3	1.1
Concomitant non-opioid analgesic treatments	
Pregabalin	21.2
Gabapentin	12.7
Paracetamol	45.8
NSAIDs	15.8
Duloxetine	16.3
Topiramate	1.4
Triptan	3.0
Nefopam	5.7
Amitriptyline	19.5
Clomipramine	2.6
Carbamazepine	0.8
Others	27.3
Number of overall analgesic treatments	
1	11.9
2	26.4
≥3	61.7
Benzodiazepines	38.4
Comorbidities	
Depression (/10)	5.7 ± 2.5
Score <5/10 (moderate to severe depression)	29.0
Anxiety (/10)	5.0 ± 2.7
Score ≥5/10 (moderate to severe anxiety)	59.9
Sleep quality (/10)	4.4 ± 2.6
Score <5/10 (poor quality)	54.1
Opioid use disorders	
Craving	6.9
Overuse*	42.5
Use for non-analgesic effects	30.9
Withdrawal symptoms	65.7

### Primary objective–prevalence of prescription opioid misuse

**FIGURE 2:**
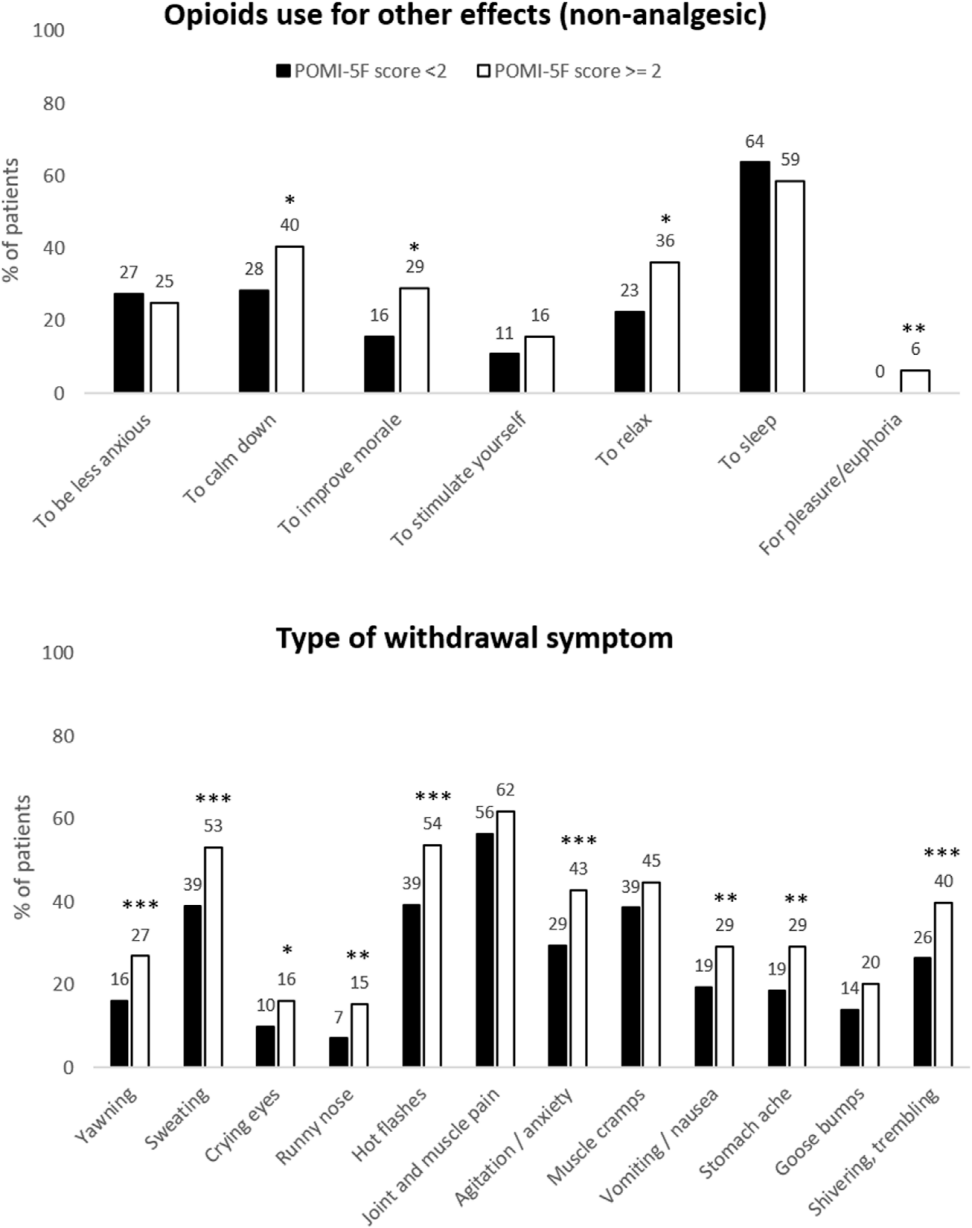
Type of opioid use for effects other than analgesia, and type of withdrawal symptoms. Data of all included patients (*n* = 951) are presented as a bar chart with associated percentages. Misusers (POMI-5F score ≥2) are in white bars and non-misusers (POMI-5F score <2) are in black bars. *p < 0.05; **p < 0.01 and ***p < 0.001.

Among the 951 patients with CNCP, 422 (44.4%, 95% CI = 41.2–47.6) reported a POMI-5F score ≥2, meaning the presence of POM. The majority reported having withdrawal symptoms when stopping or delaying opioid treatment (81.5%), as well as taking higher doses than prescribed for lack of sufficient pain relief (72.4%). In addition, 45.5% of them reported taking their opioid treatment for something other than pain relief. The different purposes of using their opioids for non-analgesic effects, correlated with the presence of a POM, was to improve mood and to calm-down/to relax (see Figure 2 for details). Lastly, 13% of them reported craving behavior for their opioid.

### Secondary objectives–Exploratory analyses

Characterization of the participants was performed according to the POMI-5F score (<2 versus ≥2). Our results from univariate analysis highlighted certain factors that could be associated with POM: younger patient, male sex (among men, 50.2% had a POM, and among women, 41.9% had POM, *p* = 0.02), with professional activity, suffering from severe nociplastic pain, depressive and sleep disorders (Tables 2, 3). The use of codeine and benzodiazepines was correlated with the presence of POM as well as having more than one opioid prescribed. Finally, no relationship was found between the presence of POM and other concomitant analgesic treatments. The use of strong opioids does not seem to be more at risk of developing misuse than weak opioids.

**TABLE 2:** Comparison between characteristics of non-misusers and misusers.

	POMI-5F score <2 (*n* = 529)	POMI-5F score ≥2 (*n* = 422)	*p*-value	Effect size [95%CI]
Sociodemographic characteristics				
Female sex	72.1	64.9	**0.020**	0.08 [0.01; 0.14]
Age (years)	54.0 ± 13.8	49.1 ± 11.4	**<0.001**	0.39 [0.26; 0.52]
Single	33.3	36.1	0.406	−0.03 [−0.09; 0.04]
Professional situation				
Student	1.2	1.2	**<0.001**	0.20 [0.14; 0.26]
Unemployed	9.1	13.8		
Retired	27.6	11.7		
Employed	62.2	73.3		
In activity	53.1	46.7	0.161	0.07 [0.01; 0.15]
Work stoppage	14.3	18.5		
Disability	41.0	44.4		
Opioid use disorders				
Craving	2.1	13.0	**<0.001**	0.21 [0.16; 0.26]
Overuse*	18.5	72.4	**<0.001**	0.54 [0.49; 0.60]
Withdrawal symptoms	53.1	81.5	**<0.001**	0.36 [0.30; 0.42]
Use for non-analgesic effects	19.3	45.5	**<0.001**	0.28 [0.22; 0.34]
Pain characteristics				
Type of pain				
Neuropathic	47.0	43.4	0.274	−0.04 [−0.10; 0.03]
Nociplastic	58.4	71.2	**<0.001**	0.13 [0.07; 0.19]
Nociceptive	36.0	25.9	**0.001**	−0.11 [−0.17; −0.04]
Pain duration				
6–12 months	2.3	2.9	0.404	0.04 [−0.01; 0.10]
1–5 years	36.3	32.3		
>5 years	61.4	64.8		
Pain intensity (/10)	5.7 ± 1.8	6.1 ± 1.8	1.000	−0.22 [−0.35; −0.09]
Mild (0–3)	12.2	7.6	**<0.001**	0.14 [0.08; 0.20]
Moderate (4–6)	55.1	47.1		
Severe (7–10)	32.6	45.3		
Pain relief by treatment (%)	50 [35; 70]	50.0 [40; 70]	0.844	−0.07 [−0.20; 0.06]
Treatments				
Opioids				
Morphine	13.8	15.6	0.460	0.03 [−0.04; 0.09]
Fentanyl	7.4	7.3	1.000	0.00 [−0.06; 0.06]
Oxycodone	18.5	21.8	0.221	0.04 [−0.02; 0.10]
Hydromorphone	0.6	0.7	1.000	0.01 [−0.05; 0.07]
Tramadol	46.3	41.5	0.148	−0.05 [−0.11; 0.01]
Codeine	11.7	18.2	**0.005**	0.09 [0.03; 0.16]
Dihydrocodeine	1.9	2.4	0.654	0.02 [−0.05; 0.08]
Opium	12.1	13.3	0.624	0.02 [−0.04; 0.08]
Weak opioids**	57.5	57.3	1.000	0.00 [−0.07; 0.07]
Strong opioids***	47.3	52.4	0.130	0.05 [−0.01; 0.11]
Number of concomitant opioids				
1	88.5	81.0	**0.003**	0.11 [0.05; 0.17]
2	11.0	17.3		
≥3	0.6	1.7		
Benzodiazepines	32.9	45.0	**<0.001**	0.12 [0.06; 0.19]
Comorbidities				
Depression (/10)	5.9 ± 2.5	5.5 ± 2.4	**0.005**	0.17 [0.04; 0.30]
Score <5/10 (moderate to severe depression)	26.2	32.4	**0.043**	−0.07 [−0.13; 0.00]
Anxiety (/10)	4.8 ± 2.7	5.3 ± 2.6	0.990	−0.16 [−0.29; −0.03]
Score ≥5/10 (moderate to severe anxiety)	57.9	62.5	0.159	0.05 [−0.02; 0.11]
Sleep quality (/10)	4.6 ± 2.6	4.1 ± 2.6	**0.005**	0.17 [0.04; 0.30]
Score <5/10 (poor quality)	52.4	56.3	0.262	−0.04 [−0.10; 0.02]

Data of patients with a POMI-5F score <2 (non-misusers; *n* = 529) and patients with a POMI-5F score ≥2 (misusers; *n* = 422) are presented as percentages, mean ± standard deviation or median (25th; 75th percentiles). * Dosage or frequency higher than prescribed; ** Tramadol, Codeine, Opium, Dihydrocodeine; *** Morphine, fentanyl, oxycodone, hydromorphone. CI, confidence interval.

Statistically significant values (*p* < 0.05).

**TABLE 3:** Factors related to the presence of prescribed opioid misuse. Data are presented as Odds-ratios and 95% confidence interval (CI).

	Odds ratio	(95% CI)	*p*-value
Mild pain	*REFERENCE*		
Moderate pain	1.64	(0.96; 2.81)	0.070
Severe pain	2.31	(1.34;4.00)	0.003
Craving	3.69	(1.76; 7.75)	0.001
Use for non-analgesic effect	2.37	(1.76;3.33)	<0.001
Withdrawal symptoms	3.12	(2.22;4.39)	<0.001
Nociplastic pain	1.62	(1.17;2.25)	0.003
Codeine	1.72	(1.12;2.64)	0.014
Benzodiazepines	1.59	(1.17;2.18)	0.003
Male sex	1.52	(1.08;2.14)	0.017
≥60 years	*REFERENCE*		
50–59 years	2.14	(1.39;3.29)	0.001
<50 years	2.21	(1.46;3.33)	<0.001

From an exploratory perspective, we performed a multivariable analysis to determine associated factors with the presence of POM (Table 3) and a cluster analysis to determine the profile of POM patients (Table 4). Multivariable analysis was conducted for 868 patients (868/951 = 91.3%) and showed that severe pain, nociplastic pain (e.g., fibromyalgia), young age (<50 years), male sex, codeine prescription, and co-prescription of benzodiazepines were associated with the presence of POM. Logically, this association was also observed in the presence of OUD (craving, use for non-analgesic effects, withdrawal symptoms). Comparison of our selected population for the multivariate analysis with the unselected population (*n* = 83) shows almost similar populations with a slightly higher proportion of women (*p* = 0.037) and a slightly lower proportion of neuropathic pain (*p* = 0.034) in the multivariate analysis population (data not shown). The MCA of the POM population was conducted for 377 patients (377/422 = 89.3%) who had no missing data for the variables included in the analysis, and 45 were removed. These two samples were similar in all assessed criteria (data not shown). The MCA showed the presence of three clusters of patients. The clusters differed mainly in age, type of pain, pain intensity, and type of opioid (weak or strong). Clusters 1 and 3, which were quite similar (sex, age, profession), differed from each other by the type of pain and their opioid treatment. Cluster 1 included mostly patients with nociplastic pain and weak opioids, whereas cluster 3 included mostly patients with different types of chronic pain and treated with strong opioids. We also noticed that cluster 3 tended to have more depressive disorders and co-prescription of benzodiazepines (marginal difference, *p* = 0.09). Cluster 2 differed from the other clusters in that it corresponded to elderly, retired patients with mild to moderate various chronic pain.

**TABLE 4:** Characteristics and comparison of subgroups of misusers (cluster analysis).

	Cluster 1 (*n* = 179)	Cluster 2 (*n* = 43)	Cluster 3 (*n* = 155)	*p* value
Sociodemographic characteristics				
Female sex	71.5	62.8	61.3	0.125
Age (years)				
<44	28.5	0.0	**37.4**	**<0.001**
45–51	29.6	0.0	31.6	
52–59	**39.7**	0.0	26.5	
≥60	2.2	**100.0**	4.5	
Profession				
Employed	**82.1**	0.0	**83.2**	**<0.001**
Retired	0.6	**97.7**	0.0	
Unemployed	17.3	2.3	16.8	
Pain characteristics				
Type of pain				
Neuropathic	29	51.2	54.2	**<0.001**
Nociplastic	**78.8**	65.1	59.4	**0.001**
Nociceptive	23.5	11.6	31.0	**0.027**
Pain intensity				
Mild (0–3)	8.4	**20.9**	4.5	**0.028**
Moderate (4–6)	48.0	44.2	47.7	
Severe (7–10)	43.6	34.9	47.7	
Opioids				
Morphine	1.7	23.3	27.7	**<0.001**
Oxycodone	1.7	23.3	43.9	**<0.001**
Tramadol	71.5	46.5	5.8	**<0.001**
Codeine	33.0	9.3	5.8	**<0.001**
Opium	3.9	7.0	26.5	**<0.001**
Fentanyl	1.1	9.3	13.5	**<0.001**
Weak opioid*	**100.0**	53.5	12.3	**<0.001**
Strong opioid**	8.4	53.5	**100.0**	**<0.001**
Benzodiazepines	40.2	44.2	52.3	0.087
Comorbidities				
Anxiety				
Score ≥5/10 (moderate to severe anxiety)	63.1	58.1	64.5	0.75
Depression				
Score <5/10 (moderate to severe depression)	25.1	32.6	**38.7**	**0.29**
Sleep quality				
Score <5/10 (bad quality)	55.3	46.5	60.6	0.23
Opioid use disorders				
POMI score	3.04 ± 1.04	2.70 ± 0.89	2.94 ± 1.00	0.137
Craving	14.0	14.0	12.3	0.891
Use for non-analgesic effect	48.6	46.5	43.9	0.688
Overuse***	73.2	62.8	73.5	0.346
Withdrawal symptom	81.0	72.1	86.5	0.078

Data are presented as percentages or mean ± standard deviation. Values of interest distinguishing the clusters are shown in bold. * Tramadol, codeine, opium, dihydrocodeine; ** Morphine, fentanyl, oxycodone, hydromorphone; *** Dosage or frequency greater than prescribed. In bold: main characteristics of cluster and different from other clusters.

Statistically significant values (*p* < 0.05). Bolded values represent values of interest for differences between clusters.

## Discussion

Currently, only the POMI scale (Knisely et al., 2008) is used to specifically assess POM and can easily be used in clinics. Nevertheless, many studies assessing OUD have used other non-specific scales, such as the DSM-5, which assessed addiction, dependence and OUD without distinction so it may confuse and misestimate the rate of POM(Kaye et al., 2017). In addition, there is a real challenge in terms of clinical management to distinguish “aberrant use” of prescribed opioids from addiction to these substances in chronic pain. It does not seem relevant or applicable to directly transpose directly the addiction diagnoses to CNCP patients. In this study, we updated the prevalence of POM in French patients with CNCP, using the recently translated and validated POMI-5F scale (Delage et al., 2022), specifically adapted to our French patient population.

The prevalence of POM, obtained from 951 patients followed-up in pain clinics and addictology centers, was 44.4%. No study has specifically assessed the prevalence of POM with the POMI scale, making it difficult to compare our results with the literature. Nevertheless, two French studies assessing OUD with the DSM-5 (not validated in French) showed a prevalence from 43% to 52% (Eiden et al., 2019; Guillou-Landreat et al., 2021), 26.5% in a German study (Just et al., 2019) and 34.9% in an American study (Boscarino et al., 2011). The study of Guillou-Landreat *et al.* (Guillou-Landreat et al., 2021) also assessed POM with the POMI scale but provide no information on the percentage of POM. The reasons for the variability of prevalence between studies may be the differences in population, the scale used and methodology. Unlike other studies, our study was multicenter with pain clinics and addictology centers throughout France, with large number of patients, various types of CNCP, various physician specialties, and using a French-validated scale specifically assessing POM–POMI-5F (Delage et al., 2022).

Concerning the characteristics of our included patients, we have similarities in terms of sex ratio (about 70% female), age (about 50 years old), and marital status (about 35% single) with the studies mentioned previously. It is noteworthy that in the German study, the patients were older (61.8 years old), and we have fewer unemployed patients (32.9%) compared to the studies of Eiden et al. (2019) (54.5%) and Boscarino et al. (2011) (74.3%). These differences may also explain our higher prevalence as it has been shown that patients with OUD were younger (Boscarino et al., 2011) and in employment (Delage et al., 2022). Concerning CNCP, related-comorbidities and treatment characteristics, some differences exist between studies. We have a majority of neuropathic and nociplastic pains with a severe pain intensity, like Eiden *et al.* study (Eiden et al., 2019). The study by Just et al. (2019) had mainly back pain (considered as neuropathic or mixed pain), while the other studies did not detail the type of pain. The minimum duration of CNCP was 6 months in our study, ranging from a minimum of 3–12 months in the other studies. In our patients, the most prescribed opioid treatment was tramadol, followed by oxycodone, and the minimum duration of opioid treatment was 3 months. Our result is similar to that of Eiden et al. (2019) while other studies did not provide enough detail on the opioid treatments used. Lastly, our patients suffered from anxiodepressive and sleep disorders, a comorbidity that was little or not evaluated in the other studies.

Concerning the characteristics of our patients considered as misusers (POMI-5F score ≥2), we were able to determine POM-related factors and profiles using multivariable analysis. The results obtained show that young male patients, patients suffering from severe nociplastic pain, codeine prescription and a co-prescription of benzodiazepine were associated with a POM. These characteristics are also mainly found as associated factors for misuse, addiction and/or OUD in the literature (Boscarino et al., 2011; Lakha et al., 2014; Just et al., 2018, 2019; Campbell et al., 2020). Interestingly, codeine prescription has been shown to be associated to the presence of POM, as previously demonstrated (Roussin et al., 2013; Van Hout and Norman, 2016; Nielsen and Van Hout, 2017; Perelló et al., 2021), whereas tramadol has not despite several studies having shown this association (Atluri et al., 2014; Dart et al., 2015; Chenaf et al., 2016). Other differences exist with the literature, such as the association between oxycodone use and OUD but not codeine (Guillou-Landreat et al., 2021), employment status were not related to OUD (Boscarino et al., 2011), sex, age and depressive disorder were not related to OUD, pain intensity was not related to OUD (Campbell et al., 2020), and type of pain, pain intensity, and sleep disorders were not related to OUD (Lakha et al., 2014). These differences could be explained by the methodologies used to assess OUD/POM (POMI versus DSM-5, Opioid Risk Tool [ORT] and COMM scales), the definition of chronic pain (duration of pain), and the study population (primary care, pain clinics, addictology centers), which vary considerably between studies and countries according to clinical practices.

Lastly, we were able to separate our POM population into three subgroups differentiated mainly by age (cluster 2 = patients aged +60 years) and type of opioid (cluster 1 = weak opioids; cluster 3 = strong opioids). Interestingly, the clusters did not differ on the POMI-5F score and OUD symptoms (craving, withdrawal symptoms, use for non-analgesic effects, and overuse). Moreover, there was no difference in the intensity of misuse (POMI score) between the weak versus strong opioid cluster (1 and 3 respectively). This reinforces the fact that the distinction between weak and strong opioids is not appropriate for use disorders, in line with the recent recommendations of the French National Health Authority on opioid drugs (HAS, 2022) (Bon usage des médicaments opioïdes: antalgie, prévention et prise en charge du trouble de l’usage et des surdoses). There was also an association between the presence of depressive disorders and strong opioid use (cluster 3), with cluster 1 on weak opioids having significantly less depression. This observation is in line with the literature which indicates a link between strong opioids and depressive disorders (Martins et al., 2012). Our three clusters also seem to partially reflect the French management recommendations for chronic pain patients on opioids, with the use of weak opioids for nociplastic pain (cluster 1), such as fibromyalgia, and strong opioids as third-line treatment for chronic pain (cluster 3) (Moisset et al., 2016). Finally, our results suggest that sociodemographic characteristics, type of pain and treatment were not associated with the intensity of POM.

### Limitations

Our study has certain limitations, such as a measurement bias, which occurs frequently in observational studies (Althubaiti, 2016). Nevertheless, self-reporting permits a wider range of responses than many other data collection designs (Zhu et al., 1999). Measurement bias can arise from recall period, selective recall, social desirability, or sampling approach. In our study, the recall period and social desirability might be the major risks (Althubaiti, 2016). Since all the questions dealt with the present moment or, at the latest 2 weeks earlier, the recall bias can be considered negligible. Self-reporting data can also be affected by an external bias caused by social desirability or approval, especially in cases where anonymity and confidentiality cannot be guaranteed at the time of data collection (the patient may be reluctant to mention misuse to his/her prescriber). This is especially true when questions are asked about non-recommended practices, which can lead to feelings of guilt or shame on the part of the patient towards their physician. Therefore, we cannot exclude the fact that some patients may have minimized their POM, and thus underestimated the prevalence of POM in our study. Another major limitation is selection bias. Indeed, only French patients followed in pain clinics and addictology centers were included. This selection bias does not allow us to generalize our results to all CP patients, but only to the French population of CP patients followed up in pain and addictology centers. The last limitation is the non-inclusion of patients with chronic cancer pain (CCP). Therefore, we cannot state the prevalence of POM for this type of patient. There are several reasons for not including these patients. In France, the management of patients suffering from CCP is quite different from that of other chronic pains. Also, these patients are not or only rarely seen in pain clinics and addictology centers, but rather in anticancer centers. Furthermore, 90% of patients on strong opioids in France do not have cancer and 70% of opioid prescriptions are for CNCP(Chenaf et al., 2019; ANSM, 2021). Moreover, the French guidelines (Moisset and Martinez, 2016) recommend limiting the prescription of opioid analgesics to 3 months in CNCP, while there is no recommended limit for CCP. Finally, it seems that patients with CCP had a lower addiction rates (Højsted and Sjøgren, 2007). It is therefore in the population of patients with CNCP that the use of opioids for more than 3 months may pose a problem and raise the question of POM.

## Conclusion

The updated prevalence of POM in French patients with CNCP remains high, with almost half of the patients concerned. In view of all our results, certain factors should draw the attention of practitioners to the profile of patients at risk of POM, such as: young age, male, suffering from severe nociplastic pain, having a codeine prescription and a co-prescription of benzodiazepine. Nevertheless, because of the cross-sectional design of this study, further longitudinal studies are required to assess the findings. Our study also highlights that distinguishing between strong and weak opioids is not appropriate in the context of use disorder. Finally, we encourage French-speaking practitioners to use the POMI-5F scale to assess the presence of OUD in their patients receiving opioid-based therapy.

## POMA network

Debbah Abdelouahab, Peyre Alexandre, Simon Anna, Defeuillet Catherine, Wiart Catherine, Sureau Christophe, Vulser Cristofini Claire, Bouhassira Didier, Touchard Emmanuelle, Collin Elisabeth, Serra Eric, Perez-Varlan Evelyne, Mohy Frédérique, Peyriere Hélène, Le Borgne Jean-Marie, Poinsignon Jean Paul, Micallef Joëlle, Dy Lénaïg, Amilhaud Marlène, Venard Maria, Dorsner-Binard Marie, Berrier Oui Marie, Martial Maud, Feuillet Maryline, De Rijk Pablo, Ginies Patrick, Kieffert Patrick, Giraud Pierric, Aerts Raluca, Le Boisselier Reynald, Cauchin Sonia, Pouplin Sophie, Corand Virginie, Perier Yannick, Poujol Yves.

## Data Availability

The raw data supporting the conclusion of this article will be made available by the authors, without undue reservation.
